# The effect of residential environment satisfaction on depression in the elderly: Focusing on the mediating effect of stress

**DOI:** 10.3389/fpubh.2022.1038516

**Published:** 2022-10-04

**Authors:** Ji Hwan Park, Jeong Min Choi

**Affiliations:** ^1^Department of Landscape Architecture, Mokpo National University, Muan County, South Korea; ^2^Department of Social Welfare, Mokpo National University, Muan County, South Korea

**Keywords:** residential environment satisfaction, stress, elderly, mediating effect, depression

## Abstract

This study aimed to determine the mediating effect of stress on the relationship between residential environment satisfaction and feelings of depression in the elderly. To achieve the purpose of the study, the researcher personally conducted interviews with 250 senior citizens residing in Jeollanam-do, South Korea, from October to November 2019. SPSS version 27.0 and Hayes' PROCESS ver. 4.0 were used for data analysis. As a result of testing the research hypothesis, a partial mediating effect of stress on the relationship between residential environment satisfaction and feelings of depression was confirmed. Based on these results, diverse practical and policy suggestions were recommended. First, a connection between barrier-free walking and public transportation linking elderly residences and major living facilities (transportation facilities, medical facilities, cultural facilities, social welfare facilities, parks, etc.) was suggested. Second, the application of a universal design was proposed when remodeling or building elderly residences. Third, policies promoting social participation of the elderly and providing assistance to increase the intimacy of their relationship with family members and neighbors are necessary to enable older adults to maintain their social relationships. Fourth, programs, such as educational schemes that provide older adults with a greater understating of stress, must be developed in parallel to facilitate stress self-management interventions. In future, it is necessary to include additional mediating or moderating variables to generalize these findings to the larger population.

## Introduction

The increasing elderly population is a universal and challenging phenomenon, and among all countries, elderly population in Korea is increasing the fastest. According to the Ministry of Health and Welfare and Korea Institute for Health and Social Affairs (2017), as of 2020, 15.7% of elderly population in Korea aged 65 and over has entered an aging society, and 20.3% is expected to enter a super-aging society in 2025. Specifically, results have suggested that 49.6% of all Korean households will be elderly households by 2047 (1).

Various social and psychological/mental issues are emerging as a result of this trend, with depression being one of the most common consequences. According to the Ministry of Health and Welfare and Korea Institute for Health and Social Affairs (2021), 13.5% of the Korean elderly population showed symptoms of depression in 2020 (2). According to the congresswoman Kang Seon-Woo, who reconstructed the data from the Health Insurance Review and Assessment Service, the number of elderly individuals experiencing depressive episodes and recurrent depressive disorder increased by 58% from 195,648 to 309,749 between 2010 and 2018 (3). This figure amounts to a nearly four-fold increase in the cases of major depressive disorder diagnosis compared with other adults. In addition, the proportion of elderly exhibiting depressive symptoms, yet without a diagnosis of depressive disorder, amounts to 15% of the total elderly population (4). Such results indicate that the prevalence of depression in the elderly population is significantly higher than the actual statistical data.

Depression in the elderly leads to personal and social loss. According to the National Health Insurance Service (2011), medical expenses of depression in the elderly increased by 123.4% between 2004 (KRW 29.5 billion) and 2009 (KRW 65.9 billion) (5). In addition, the National Institute of Mental Health (2021) highlights that depression can cause and/or exacerbate various diseases, such as cancer, heart disease, chronic pain, and diabetes (6).

Therefore, the Korean government is currently taking various measures to mitigate depression in the elderly by establishing metropolitan mental health promotion centers. Moreover, several research studies conducted by academic institutions have been analyzing that these factors increase the risk of depression in the elderly. As a result, community variables that increase depression have been gaining significant attention in recent years. In particular, the existence of a healthy and age-friendly environment, including both residential and social environmental variables, is of greater importance to the elderly than to the youngsters because of aging-related issues, such as social isolation and physical deterioration. In 2007, the World Health Organization (WHO) advocated the development of the concept of age-friendly cities, emphasizing the importance of a healthy residential environment for the elderly. A recent report by the National Institute of Mental Health (2021) also pointed out that genetic, biological, psychological, and environmental factors can play an essential role in causing depression (6).

Previous studies have also supported the significance of the relationship between residential environment satisfaction and feelings of depression (7, 8). The Environmental Press Theory explains that environmental pressure induces negative emotions in the elderly (9). Despite these findings, previous studies have not adequately explained the context of the relationship between residential environment satisfaction and depression. Koo and Chai (10) indicated that while studies investigating the direct effect of residential environment satisfaction on depression are being conducted, those analyzing the various mechanisms between the two variables are still lacking (10). In fact, Koo and Chai (10) assumed that stress should be considered the third variable in the relationship between residential environment satisfaction and depression (10).

Consequently, this study focuses on stress, which can be defined as a bodily response to the residential environment of the elderly (11, 12). When stress is increased to unbearable levels, depression increases. According to the Stress Exposure Model, continuous exposure to stress will clinically induce major depressive disorder in an individual (6, 13).

Based on previous studies, stress may be estimated to play the third important role in the relationship between residential environment satisfaction and depression in the elderly. According to the hypothesis proposed by Cutrona et al. (14), the environmental variable increases depression, with everyday stress as a mediator (14). Furthermore, previous studies support the significant relationships between residential environment satisfaction and stress (15, 16) as well as between stress and depression (17, 18).

Thus, the present study aimed to verify the mediating effect of stress on the relationship between residential environment satisfaction and depression. Through this study, we intended to present practical and policy suggestions for the management of depression in the elderly.

## Literature review

### Residential environment and depression

Socially, mental health is an important issue (19–21). The main issue of these mental health problems is depression. Depression is one of the emotions that people experience in daily life. Most sad emotions will disappear after a while. However, the problem is that the persistence of depressive symptoms can lead to the development of mental illness (6). Symptoms of depression appear to be different between the elderly and other adults. A distinct characteristic of depression in the elderly is the manifestation of depressive symptoms, such as concentration disorders, memory disorders, and health anxiety, without any direct complaints (4). According to a study performed by the Ministry of Health and Welfare & Korea Institute for Health and Social Affairs (2017), as of 2017, 21.1% of the elderly population in Korea exhibited symptoms of depression (1).

Among the causes for the increasing rates of depression in the elderly, residential environment satisfaction has recently attracted significant attention in academia. Residential environment is used in various concepts, including residential satisfaction and housing satisfaction, but considering that these concepts show similar patterns, this study has conceptualized them as residential environment satisfaction (8). And Residential environment is the space within a facility building that forms the basis of human life, and when satisfaction within these spaces is high, residential environment satisfaction is also said to be high (7). The importance of residential environment is also reflected by the fact that it is stated as one of the main needs in Maslow's Hierarchy of Needs. However, as of 2019, residential environment satisfaction in Korea's elderly decreased from 2.91 points in 2017 to 2.87 points in 2019. Particularly, residential environment satisfaction has been found to be lower in the elderly than in other households (22).

Residential environments influence various aspects. The Environmental Pressure Theory provides a major perspective on the residential environment, which explains that pressure from a neighborhood environment influences the elderly and induces negative emotions (9, 23). Hence, it can be deduced from this theory that the residential environment has a significant effect on the development of depressive symptoms. In fact, previous studies also support the relationship between residential environment satisfaction and depression. According to a study by Lee and Kim (7), the physical residential environment, as perceived by the elderly themselves, has a negative effect on depression (7). Also, according to a study by Koo and Chai (10), depression in the elderly population decreases with increasing residential environment satisfaction while controlling gender, age, educational level, and marital status (10). Baik's (8) study also highlighted that residential environment satisfaction has a negative effect on depression while controlling for variables such as cohabitation with family (8).

Based on these findings, it can be suggested that residential environment satisfaction has a significant impact on depression.

### The mediating effect of stress

Stress is a bodily response, implying a disturbance in homeostasis, to certain events and situations. Depending on the situation and on individual traits, some individuals may experience stress while others may not. In other words, the response to stress varies for each individual (11, 12, 24).

Although there are several well-established factors that cause stress, Eredoro and Egbochuku (12) indicated that environmental factors, such as transportation, poor housing, and pollution, may also play a major role in stress (12). Previous studies also suggested that there is a distinct connection between residential environment satisfaction and stress. According to Jin and Jang (15), among respondents residing in dormitories, students who had low levels of satisfaction with their physical living environment experienced more personal stress (14). Also, students with low levels of satisfaction with their social and psychological residential environment were more perceptive to environmental stress. Fornara et al. (16) indicated that satisfaction with the space at home has a significant effect on perceived stress (15).

Meanwhile, according to the Stress Exposure Model, factors such as residential environment increase stress. Also, continuous exposure to acute or chronic stress may induce or cause a relapse of clinical major depressive disorder (6, 13). Previous studies also support the significance of the relationship between stress and depression. According to a study by Shin and Kim (17), stress in elderly women had a significant effect on depression. In particular, the explanatory power of stress for depression has been reported to be 26% (17). Park's (18) study reported that stress affects depression (18).

Altogether, the results of the previous studies presented above underline that residential environment satisfaction influences the development of depressive symptoms in the elderly through stress.

## Research method

### Research model

**Figure 1:**
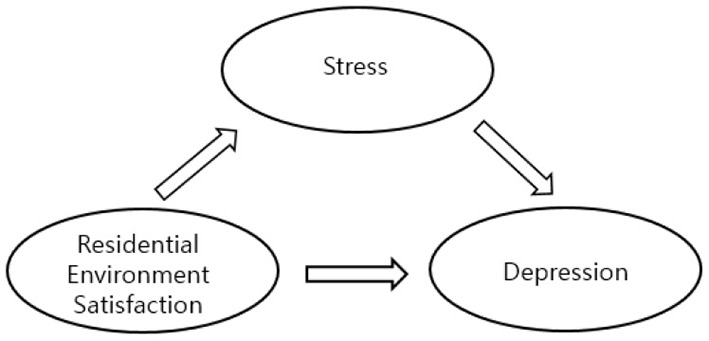
Research model.

This study aimed to examine the mediating effect of stress on the relationship between residential environment satisfaction and depression, and a research model was established, as shown in Figure 1.

### Research subjects and methods

A total of 250 elderly individuals living in Jeollanam-do province in South Korea were included in this study. The survey period was from October to November 2019, and the researcher personally visited and conducted all interviews. Data analysis was performed using SPSS version 27.0. First, the characteristics of major variables were identified using frequency and descriptive statistical analysis. Second, a correlation analysis was performed to investigate the relationship between the variables and the problem of multicollinearity. Third, the significance of the mediating effect of stress on the relationship between residential environment satisfaction and depression was confirmed using Hayes' PROCESS ver. 4.0.

### Measurement tools

For depression, Kee's Korean version of the Geriatric Depression Scale Short Form for the elderly was used (25). The depression scale consisted of 11 items, and the responses were rated on a 4-point Likert scale ranging from “strongly disagree” (1 point) to “strongly agree” (4 points). The positive items were reverse scored with negative items; thus, higher scores indicated higher levels of depression. The reliability of the scale was confirmed with Cronbach's alpha coefficient of 0.855.

The scale used by Lee (26) was also employed in this study to quantify residential environment satisfaction (26). The residential environment satisfaction scale consisted of 16 items, and the responses were rated on a 4-point Likert scale ranging from “strongly disagree” (1 point) to “strongly agree” (4 points). Therefore, higher scores indicated higher levels of residential environment satisfaction. The reliability of the scale was confirmed with Cronbach's alpha coefficient of 0.855.

Stress was measured using the Lee's instrument (27). The stress scale consisted of a single item, and the response was rated on a 1–10-point Likert scale. Here too, higher scores indicated higher stress levels.

The control variables used in this study were sex, age, educational level, and family form, and the following were treated as dummies: sex, 0 = women and 1 = men; education, 0 = uneducated and 1 = elementary school or higher; family form, 0 = single family and 1 = cohabiting with family.

## Analysis results

### Demographic characteristics

The demographic characteristics of the respondents are presented in Table 1. As shown, 41.6% were men and 58.4% were women, of whom 30.8% were in their 60's, 47.6% in their 70's, and 21.6% in their 80's or older. With respect to the respondents' educational level, 26.4% were reported as uneducated and 73.6% as having education above the elementary level. As for the family form, 26.4% of the responders were single and 73.6% were cohabiting with their families.

**Table 1:** Demographic characteristics (*N* = 250).

**Classification**		**Frequency**	**Percentage**
Sex	Male Female	104 146	41.6 58.4
Age	60's 70's 80's or older	77 119 54	30.8 47.6 21.6
Education level	Uneducated Elementary school or above	66 184	26.4 73.6
Family form	Single Cohabiting with family	66 184	26.4 73.6

### Descriptive statistics of main variables

The results of the descriptive statistical analysis of the key variable are shown in Table 2. First, residential environment satisfaction was found to be 2.56 points (SD = 0.46) on a scale of 1–4. Stress was reported to be 4.51 points (SD = 2.30) on a scale of 1–10. Finally, depression was confirmed by a score of 2.30 (SD = 0.40) on a scale of 1–4.

**Table 2:** Descriptive statistics of the main variables.

**Variable**	**Score range**	**mean**	**SD**
Residential environment satisfaction	1–4	2.56	0.46
Stress	1–10	4.51	2.30
Depression	1–4	2.30	0.40

### Correlation analysis of key variables

The results of the correlation analysis of key variables are presented in Table 3. A significant relationship was found between depression and residential environment satisfaction, at r = −0.400 and r = 0.509 with stress. The correlation between residential environment satisfaction and stress was −0.213, and no issues were found due to multicollinearity.

**Table 3:** Correlation.

**Variable**	**Residential environment satisfaction**	**Stress**	**Depression**
Residential environment satisfaction			
Stress	−0.213***	-	
Depression	−0.400***	0.509**	-

** p < 0.01,

*** p < 0.001.

### Verification of the mediating effect of stress

Table 4 demonstrates the relationship between residential environment satisfaction and stress. First, the model fit was found to be statistically significant at the level of F = 2.853, *p* < 0.05. Furthermore, as a result of controlling for demographic characteristics and including a variable for residential environment satisfaction, the explanatory power of stress was confirmed to be 5.5%. The level of residential environment satisfaction to stress was found to have a significant effect at the level of B = −1.151. Therefore, it can be inferred that as residential environment satisfaction increases, the stress of the elderly decreases.

**Table 4:** Relationship between residential environment satisfaction and stress.

**Classification**	**Stress**		
	**coeff**	**se**	**t**
Sex Age Educational level Family cohabitation	0.334 −0.027 −0.081 0.059	0.310 0.024 0.362 347	1.08 −1.11 −0.22 0.17
Residential environment satisfaction	−1.151	0.328	−3.51***
constant F R^2^		9.310 2.853* 0.055	

* p < 0.05,

*** p < 0.001.

Dummy: sex (1= male), education level (1 = elementary school or above), family form (1 = cohabiting with family).

Table 5 demonstrates the relationship among residential environment satisfaction, stress, and depression. First, the model fit was found to be statistically significant at the level of F = 26.434, *p* < 0.001. Moreover, as a result of controlling for demographic characteristics and including residential environment satisfaction and stress variables, the explanatory power of depression was confirmed to be 39.5%. Both residential environment satisfaction and stress had a significant effect on depression at the level of B = −0.204 and B = 0.080, respectively. Therefore, depression symptoms in the elderly decrease with increasing residential environment satisfaction and decreasing stress levels.

**Table 5:** Relationship among residential environment satisfaction, stress, and depression.

**Classification**	**Depression**		
	**coeff**	**se**	**t**
Sex Age Education level Family cohabitation	−0.050 0.004 −0.088 −0.113	0.043 0.003 0.050 0.048	−1.15 1.28 −1.75 −2.33
Residential environment satisfaction	−0.204	0.047	−4.36***
Stress	0.080	0.009	8.95***
Constant F R^2^		2.310 26.434*** 0.395	

*** 6p < 0.001.

Dummy: Sex (1 = male), education level (1 = elementary school or above), family form (1 = cohabiting with family).

The significance of the mediating effect of stress was verified by bootstrapping. The results of the analysis showed that stress had a significant mediating effect, as 0 was not included within the 95% confidence interval (from −0.151 to −0.037) (Table 6).

**Table 6:** Bootstrapping analysis results.

**Path of mediating effect**	**Indirect effect**	**Confidence level**	
		**LCI**	**UCI**
Residential environment satisfaction → stress → depression	−0.092	−0.151	−0.037

## Conclusion

This study aimed to examine the mediating effect of stress on the relationship between residential environment satisfaction and depression in the elderly and to confirm a partial mediating effect. The obtained result is consistent with the result of previous studies reporting that the elderly's residential environment satisfaction has a direct effect on depression (7, 8), using stress as a mediator (15–18).

Based on these results, some practical and policy implications are suggested, as follows:

First, the government should actively utilize the concept of age-friendly cities proposed by the WHO in 2007 during the designing phase of urban planning. The key to an age-friendly city is to create a residential environment that is suitable for the elderly to live. For example, accessibility to large living facilities, such as transportation facilities, medical facilities, cultural facilities, social welfare facilities, and parks, used by the elderly should be considered. To this end, barrier-free walking and public transportation linking the elderly's residences and major living facilities should be implemented (7, 28).

Furthermore, it is necessary to design residential spaces considering the lifestyle of the elderly. For example, when remodeling or building an elderly residence, a universal design should be applied. In addition, the government should expand financial support for the installation of convenience facilities for the elderly during the planning of the universal design, focusing on elderlies with low-income. Furthermore, it is imperative to pay attention to the establishment of a community-level safety net that can offer protection from crime and disasters.

Also, one of the primary needs of the elderly is aging in place (AIP) (8). To meet this need, the government has already introduced a community care policy. As suggested above, for community care to be possible, maintaining social relationships in addition to ensuring a universal design of residential spaces and a barrier-free walking environment are major policy concerns. In other words, social relationships must be maintained to enhance the residential environment satisfaction of the elderly. Thus, policies promoting social participation activities and increasing the intimacy of the relationship with neighbors and family are necessary.

In contrast, stress was confirmed to have a mediating effect on the relationship between residential environment satisfaction and depression. Therefore, policy and practical considerations are necessary to help the elderly manage stress.

According to the Environmental Pressure Theory, environmental pressure induces negative emotions in the elderly (9). Also, depression is a negative emotional response that appears as part of the process of experiencing stress in the elderly. Previous studies (6, 13) indicated that constant exposure to acute or chronic stress may induce or cause a relapse of clinical major depressive disorder.

Therefore, to prevent the decrease in residential environment satisfaction from leading to an increase in depression, it is essential to effectively manage stress, a precursor to depression. In particular, while local community health centers and social welfare institutions are providing support to older adults with depression, stress management remains relatively insufficient. Therefore, it is necessary to devise a holistic system for stress management and depression in the elderly in the future. Moreover, the development of programs, such as leisure program, educational schemes and meetings that allow the elderly to gain a greater understanding of stress, must be performed in parallel (29–34).

Despite its significance, this study has several limitations. First, this study was conducted on a partial elderly population in Jeollanam-do; thus, our findings cannot be directly generalized to a larger population. Therefore, future research should be conducted on the elderly population nationwide. In addition, despite the existence of various mediating or moderating variables in the relationship between residential environment satisfaction and depression, only the stress factor was considered in this study. Therefore, it is necessary to include more diverse mediating or moderating factors in future studies.

## Data availability statement

The raw data supporting the conclusions of this article will be made available by the authors, without undue reservation.

## Ethics statement

Ethical review and approval was not required for the study on human participants in accordance with the local legislation and institutional requirements. Written informed consent for participation was not required for this study in accordance with the national legislation and the institutional requirements.

## Author contributions

All authors listed have made a substantial, direct, and intellectual contribution to the work and approved it for publication.

## Conflict of interest

The authors declare that the research was conducted in the absence of any commercial or financial relationships that could be construed as a potential conflict of interest.

## Publisher's note

All claims expressed in this article are solely those of the authors and do not necessarily represent those of their affiliated organizations, or those of the publisher, the editors and the reviewers. Any product that may be evaluated in this article, or claim that may be made by its manufacturer, is not guaranteed or endorsed by the publisher.
